# Audio-Tactile Integration in Congenitally and Late Deaf Cochlear Implant Users

**DOI:** 10.1371/journal.pone.0099606

**Published:** 2014-06-11

**Authors:** Elena Nava, Davide Bottari, Agnes Villwock, Ineke Fengler, Andreas Büchner, Thomas Lenarz, Brigitte Röder

**Affiliations:** 1 University of Hamburg, Biological Psychology and Neuropsychology, Hamburg, Germany; 2 German Hearing Centre, Medical Clinic Hannover, Hannover, Germany; University of Montreal, Canada

## Abstract

Several studies conducted in mammals and humans have shown that multisensory processing may be impaired following congenital sensory loss and in particular if no experience is achieved within specific early developmental time windows known as sensitive periods. In this study we investigated whether basic multisensory abilities are impaired in hearing-restored individuals with deafness acquired at different stages of development. To this aim, we tested congenitally and late deaf cochlear implant (CI) recipients, age-matched with two groups of hearing controls, on an audio-tactile redundancy paradigm, in which reaction times to unimodal and crossmodal redundant signals were measured. Our results showed that both congenitally and late deaf CI recipients were able to integrate audio-tactile stimuli, suggesting that congenital and acquired deafness does not prevent the development and recovery of basic multisensory processing. However, we found that congenitally deaf CI recipients had a lower multisensory gain compared to their matched controls, which may be explained by their faster responses to tactile stimuli. We discuss this finding in the context of reorganisation of the sensory systems following sensory loss and the possibility that these changes cannot be “rewired” through auditory reafferentation.

## Introduction

Neurophysiological studies conducted in mammals have shown that multisensory neurons and the ability to integrate crossmodal information require an extensive time of sensory experience during early development in order to fully mature [1], [2]. This predicts that sensory deprivation may impair multisensory processing, as has been extensively documented in visually deprived animals [3], [4] and in blind humans too [5], [8]. In particular, [5] have suggested that there may be a sensitive period for the development of multisensory integration, after which – if adequate experience has not been made - multisensory processing will remain impaired.

The closing of these sensitive periods is in line with a regressive notion of a developmental process known as “perceptual narrowing” [6]. One of the prevailing theories of brain development is that human infants are born with the ability to sense a broader variety of stimuli than later in life [28]. Special experience available in the environment results in the functional tuning of neural systems and the emergence of expert systems. In parallel, the ability to discriminate and perceive unexplored stimuli within the same domain is lost. For example, [29] showed that within the first year of life, there is an increase in performance for the native language and a decline in non-native perception over the same period of time. The same pattern has been observed for multisensory perception too [7]. Indeed, [7] presented 4-, 6-, 8- and 10-month old infants with two side-by-side faces of a monkey producing two different species-specific calls (i.e., “coo” and “grunt”). The faces were presented either alone or concurrently with the matching call. Four- and 6-month-old infants were found to look longer at the matching audio-visual stimulus compared to the presentation of the face alone. On the contrary, older infants did not show any looking preference, suggesting the inability to match non-native faces and calls.

Both experiments suggest that perceptual narrowing shapes our perceptual expertise and that the time window for building up such expertise seems to close early in development constraining multisensory learning in adulthood.

Furthermore, it has been shown that broadly tuned basic multisensory abilities, such as intersensory synchrony, is present at birth and puts the basis for the development of other basic features of multimodal temporal experience (e.g., temporal synchrony, duration, temporal rate, and rhythm, see [31] for a review) as development progresses [6]. This suggests that atypical sensory experience may result in altered multisensory processes that require functional tuning (multisensory perceptual narrowing) and thus altered multisensory functions [23]. Indeed, a study [9] on individuals with a history of congenital cataracts which were removed between six weeks and three years employed a simple detection task for crossmodal stimuli, including combinations of audio-visual, audio-tactile and visuo-tactile stimuli. Results showed that cataract patients benefitted from redundant stimulation over unimodal stimulation (e.g., audio-visual together vs. visual alone) comparably to normally sighted and visually impaired controls, suggesting that basic multisensory processing, such as simultaneity detection, is not disrupted following congenital visual loss. In contrast, for functions, which seem to rely on multisensory, experience based tuning (i.e., higher level perceptual abilities), [5] found that congenital cataract patients did not show any multisensory gain. Indeed, these individuals were not able to improve auditory speech comprehension by reading congruent lip movements, although they were able to lipread.

The consequences of auditory deprivation have started receiving larger attention in the past twenty years (for a review see [13]), newly triggered by to the possibility of partially restoring auditory functions through a cochlear implant (CI). A CI is a surgically implanted electronic device that allows deaf individuals to perceive auditory input to some degree. Similarly as cataract patients, these individuals allow us to investigate the developmental consequences of an early or late sensory deprivation on multisensory functions (depending on age at deafness onset and age at CI implantation).

Most of the research interested in multisensory processing in CI recipients has focused on audio-visual interactions for speech comprehension, likely because the main aim of the CI is allowing recipients to communicate with other people. For example, [10] investigated the ability of late deaf CI recipients to fuse audio-visual information in the McGurk task, a classical demonstration of how visual speech can modify audition when presented with an incongruent auditory signal (e.g., presenting a spoken syllable/ba/matched with synchronous lips pronouncing the syllable/ga/, is often perceived as a third syllable:/da/). The authors found that adult CI recipients were able to fuse the incongruent crossmodal information to the same degree as controls did, in line with results of [11], who tested a group of congenitally deaf CI children on the same task. However, [11] documented audio-visual fusion only in children, who had received a CI before age 2.5 years but not in children who had received their implants later. This result suggests the existence of a sensitive period for the acquisition of audio-visual speech integration. Similarly, [12] investigated audio-visual integration for simple stimuli in early deaf CI children (i.e., who became deaf within their first year of life) who were fitted with their CI either early (between 1 and 4 years of age) or late (between 5 and 10 years of age). They employed a simple detection task, in which reaction times to auditory and visual stimuli were measured when presented alone or concurrently. The authors found that while early implanted CI children gained from cross-modal redundant stimulation similarly to hearing controls, late implanted CI children did not. These results suggest, in line with [11] that typical auditory experience within a sensitive period during the first years of life is necessary in order to develop unimpaired multisensory functions. Thus, it might be assumed that the tuning of crossmodal simultaneity detection depends on experience within the first years of life as well (note that the participants of [5] were mostly deprived for a few months only).

Overall, it appears that most studies in deaf CI recipients have investigated audio-visual integration, possibly because audio-visual integration is crucial in the context of speech perception. However, audio-tactile integration is important in many everyday situations as well, for example in the context of temporal perception, action control and object recognition [27], [30]. Furthermore, audio-tactile interactions represent another case to observe possible plastic reorganisation in the deaf brain. As has been previously shown for visually deprived individuals [3], [4], it might be that multisensory areas reorganise following auditory deprivation for the processing of visual and tactile stimuli. In other words, auditory input reaching multisensory areas after the closing of sensitive phases in development might not be able to establish contacts with neither the visual nor the tactile inputs, thus explaining the presence of impaired crossmodal interactions. For CI recipients, this would translate into showing impaired crossmodal interactions if CI implantation does not take place before the closing of sensitive phases.

To our knowledge, there is only one study that has addressed audio-tactile integration in CI recipients [15]. In this study, the authors employed an audio-tactile illusion, in which participants commonly perceive additional tactile stimuli when one touch is accompanied multiple successive sounds (see [16]). Results revealed that whereas hearing controls perceived the illusion, CI recipients did not; that is, the latter group did not show any sign of integration capability. Furthermore, the authors suggested that temporary auditory deprivation impairs multisensory integration even if deafness had not occurred at birth, since no difference was found between congenitally and late deaf CI recipients.

The findings of [15] overall suggest that prolonged deafness, irrespective of age at onset, impairs integration of audio-tactile stimuli. This is in contrast with other results showing that CI recipients with congenital onset and early surgery or late deafness are able to fuse incongruent audio-visual information ([11], [10] or are even better integrators of auditory and visual speech signals, respectively [17].

In the present study we investigated whether multisensory abilities relying on simultaneity detection develop typically or are impaired following temporary deafness acquired at different stages of development. To this aim, we tested congenitally and late deaf CI recipients, age-matched with a group of hearing controls, on an audio-tactile redundancy paradigm, in which reaction times for unisensory and crossmodal redundant signals were measured. Commonly, healthy adults show faster reaction times for redundant targets compared to unimodal stimuli [18], and this has been generally explained by coactivation models [18]. These models assume that faster reaction times for redundant signals result from an integration of the two sensory channels at some processing stage, and not by one sensory channel “winning the race” over the other (race model, [19]). [18] has proposed a race model inequality test, in which reaction times to bimodal stimulation can be assessed as deriving from integration or mere statistical facilitation (i.e., violation or non-violation of the race model, respectively).

Because the redundant target effect makes use of synchronous stimuli only, we assumed it to be the ideal paradigm to document whether CI recipients are able to integrate audio-tactile information. If experience is necessary to further shape at birth broadly tuned multisensory integration processes, we would expect only - or at least higher - redundancy gains in late deaf CI recipients with respect to congenitally deaf CI recipients.

## Method

### Participants

Ten congenitally (5 female; mean age: 25 years, range: 20–37) and ten late deaf CI recipients (4 female; mean age: 48 years, range: 23–59) participated in this study. The ten age-matched controls for the congenitally deaf CI recipients (MCCD from now on) comprised 7 females and 3 males (mean age: 24 years, range: 20–38), while the ten age-matched controls for the late deaf CI recipients (MCLD from now on) comprised 5 females and 5 males (mean age: 41, range: 20–57).

The CI recipients were recruited through local advertising and some of the late deaf CI recipients were recruited at the Medical School in Hannover (Germany). All CI recipients were profoundly deaf (>90 dB) in both ears before receiving their CI. While the congenitally deaf CI recipients became deaf at birth or immediately after, the late deaf CI recipients became deaf at different ages during development, ranging between 7 and 42 years of age. For the congenitally deaf CI recipients, implantation occurred at different ages during development, ranging between 2 and 33 years of age. For the late deaf CI recipients, age at implantation occurred in adulthood only, ranging between 19 and 54 years of age. The CI recipients had had on average 10 years of experience with their implant prior to testing (range: 1–23 years). Although the cause of deafness was unknown for most CI participants, we only recruited participants for which possibilities of cognitive impairment could be excluded (e.g., as a consequence of meningitis).

Further details about the participants, including aetiology of deafness, type of device implanted, ear implanted, age at first CI implant (i.e., in case of bilateral sequential CI, we refer to the age at which the first CI was implanted; no CI recipients was implanted simultaneously), years of experience with the CI and years of CI use are reported in Table 1.

**Table 1:** Description of CI recipients.

ID	age	gender	etiology	age at deafness onset	years of deafness	age at first implant	years of CI use	type of CI	implanted ear (side)
1	23	male	unknown	0	2	2	21	Cochlear	right
2	20	female	unknown	0	10	10	10	Advanced Bionics	bilateral
3	37	female	unknown	0	33	33	4	Cochlear	right
4	23	male	unknown	0	11	11	12	Cochlear	right
5	23	male	unknown	0	9	9	14	Advanced Bionics	right
6	23	female	unknown	0	18	18	5	Cochlear	right
7	23	male	unknown	0	14	14	9	Cochlear	right
8	23	female	unknown	0	8	8	15	Cochlear	bilateral
9	23	female	unknown	0	4	4	19	Cochlear	left
***mean***	**24** (SD: 4.6)			**0**	**12** (SD: 8.7)	**12** (SD: 8.7)	**12** (SD: 5.5)		
10	47	female	unknown	23	1	24	23	Cochlear	bilateral
11	58	male	unknown	32	21	53	5	Advanced Bionics	bilateral
12	56	female	unknown	7	43	50	6	Advanced Bionics	bilateral
13	59	female	tumor	42	12	54	5	Cochlear	bilateral
14	56	female	unknown	17	29	46	10	Clarion	bilateral
15	51	male	unknown	30	16	46	5	Med El	bilateral
16	38	female	Scarlet fever	20	13	33	5	Advanced Bionics	right
17	42	male	German measles	40	1	41	1	Cochlear	bilateral
18	23	female	unknown	18	1	19	4	Cochlear	right
19	45	male	unknown	31	1	32	13	Advanced Bionics	bilateral
***mean***	**48** (SD: 10.6)			**26** (SD: 10.4)	**14** (SD: 13.4)	**40** (SD: 11.6)	**8** (SD: 6)		

All participants had normal or corrected-to-normal vision, reported normal sensitivity to their fingertips and had no neurological impairment.

All participants provided written informed consent before being tested and received a reimbursement for their time and expenses associated with the testing, which took place in a room of the laboratory at the University of Hamburg or in one of the rooms of the Medical School of Hannover.

The study was approved by the ethical commission of the German Society of Psychology and the Medical School of Hannover. The study was conducted according to the principles expressed in the Declaration of Helsinki.

### Stimuli and Procedure

The auditory stimulus consisted of a 50 ms burst of white noise at 80 dB and was presented from two loudspeakers (Bose, Multimedia Speaker System) positioned side-by-side 60 cm in front of the participant.

Tactile stimuli were 50 ms vibrations of plastic cubes at the left index finger controlled by cell phone motors with a direct current, with a tuning frequency of 200 Hz. To ensure spatial proximity of the stimuli, participants were asked to keep their left hand close to the loudspeakers throughout the experiment. To mask the faint noise generated by the tactile stimulators, all participants wore closed headphones throughout the experiment. For CI participants, the sound pressure of the auditory stimulus was adjusted to a comfortable level.

During the experiment, participants were presented with 180 unimodal auditory, 180 unimodal tactile and 180 audio-tactile stimuli divided in two blocks and presented in random order at 1000 or 2000 ms intervals. In addition, 36 catch trials (i.e., no stimulus presented) were included to prevent participants from guessing the onset of the upcoming stimulus. The task for all participants simply consisted of pressing the space bar of the computer keyboard each time a stimulus was perceived. Trials with a stimulus, in which no response was provided, were considered as “miss”. Trials without a stimulus but with a response were counted as “false alarm”. Participants were allowed to take breaks between the two blocks. The experiment took approximately 20 minutes to complete and before the start participants were familiarized with the experiment by training them on a set of 36 trials.

### Data Analysis

We measured reaction times for all conditions (unimodal auditory, unimodal tactile and audio-tactile) for each participant. For all controls, the number of “misses” and “false alarms” was negligible (maximum of 2 for each condition). For CI participants, “misses” were on average below 3% and were not further analysed. “False alarms” were negligible (maximum 3 for each condition).

For each group, we calculated a repeated-measures ANOVA with Condition (single auditory, single tactile and bimodal stimuli) as the within-participants factor and reaction times as dependent variable. To assess the redundant target effect and reaction time differences in the different conditions we used Helmert contrasts [26].

To compare the two groups (congenitally and late deaf CI users) we ran two separate repeated-measures ANOVAS for congenitally deaf CI recipients and their matched controls, and for late deaf CI recipients and their matched controls, with Condition as the within-participants factor, Group as the between-participants factor and reaction times as the dependent variable. Furthermore, we ran independent samples t-tests to compare performance of congenitally and late deaf CI recipients to their matched controls for each condition. To assess whether the redundancy gain differed between groups, we calculated the difference between the mean reaction times in the bimodal condition and the fastest reaction time of the two unimodal stimuli with independent-samples t-test. Finally, we correlated the redundancy gain with age at deafness onset, years of deafness, age at CI implantation and years of CI use. While for the factor “age at deafness onset” the performance of all CI recipients was included, for the other factors we conducted separate correlations for each group of CI recipients.

To assess whether faster responses in bimodal trials can be explained by integration of redundant input, we used the RMITest (http://psy.otago.ac.nz/miller/Software.htm#RMITest), which tests for violations of the race model inequality described by [15] by implementing the algorithm described in [20]. More precisely, it estimates the cumulative probability distributions of the reaction times for each condition and tests whether the bimodal condition is significantly faster than the race model predicts.

In 1962, [19] proposed the well-known race model to explain why redundant targets speed up the response of most healthy participants with respect to responses to unimodal stimuli. According to this model, each signal activates a separate process that will lead one of the two to “win the race”, so that faster reaction times for bimodal stimulation end up being the expression of one of the two unimodal signals, hence to what it’s called a “statistical facilitation”.

However, [18] proposed the race model inequality (RMI), by which a violation of the inequality between redundant stimuli and the sum of unimodal stimuli should be interpreted as integration of the sensory information by neural summation (also called “co-activation mechanism”).

Therefore, for each group we tested whether the race model was violated at 10 different points (percentiles) on the cumulative distribution of their reaction times. Additionally, we compared distributions of the fastest unisensory signal and redundant targets between groups for each percentile.

Finally, to compare the amount of RMI violations between groups, we calculated the amount of violation area for each participant (computed as the difference between the minima obtained from the two unisensory signals using the method of antithetic variates and the mean reaction time in the redundant signals; see [21]), and compared the groups by means of independent samples t-tests. A negative or zero value of the violation area indicates that the amount of observed facilitation (in terms of reaction times) is likely explained by the race model; on the contrary, positive values cannot be explained by the race model. The amount of violation was also correlated to the aforementioned factors (age at deafness onset, age at implant, years of deafness and years of CI use).

## Results

One congenitally deaf CI recipient had to be discarded from the analyses because her reaction times were three standard deviations above mean. The number of MCCD was not modified because the matching participant had an age that was compatible with the mean age of the group.

**Figure 1:**
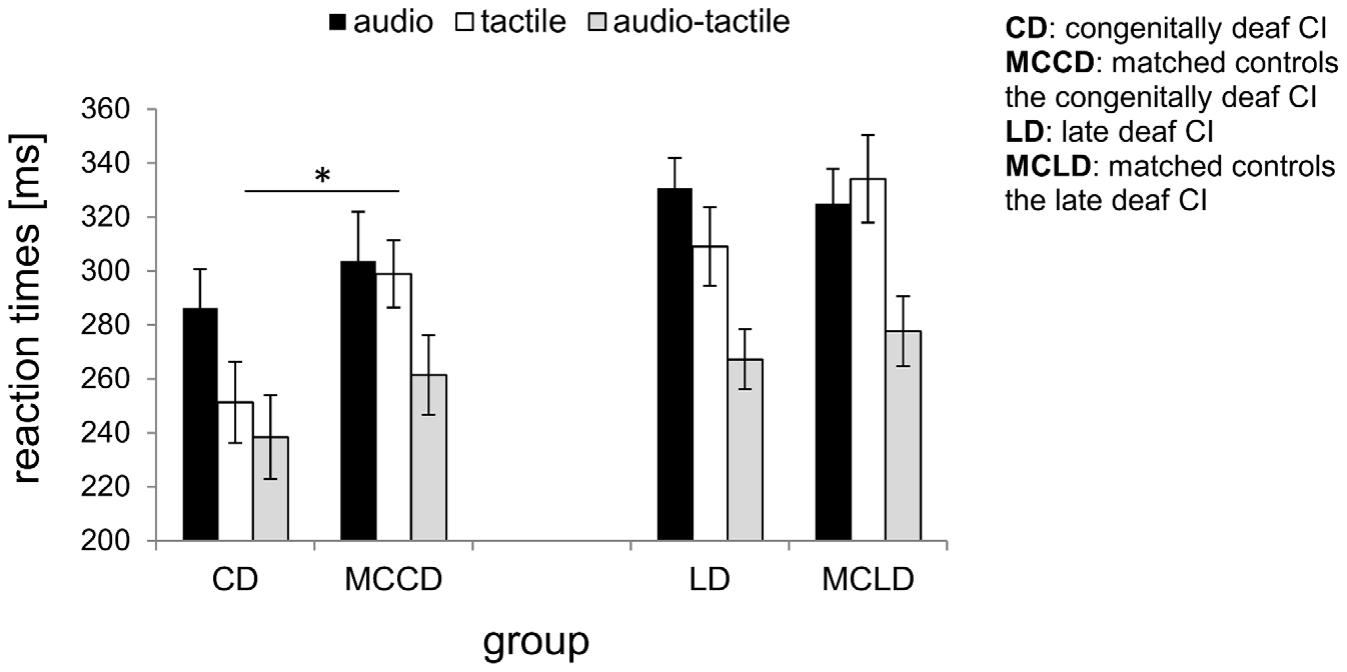
RT for unimodal auditory, unimodal tactile and bimodal. Mean reaction times (in ms, with error bars indicating the standard error) for each condition (unimodal auditory, unimodal tactile and audio-tactile), separately for the group of congenitally deaf CI recipients and their age-matched controls (left panel), and late deaf CI recipients and their age-matched controls (right panel). Note that despite all participants being faster for bimodal compared to unimodal stimuli, congenitally deaf CI recipients were faster for unimodal tactile stimuli compared their matched controls.

Figure 1 shows mean reaction times for each group in the unimodal and bimodal conditions. For each group, mean reaction times significantly differed between conditions (*congenitally deaf CI recipients*: F(1.4, 10.8) = 26.5, p<0.001, effect size η_p_^2^ = 0.8; *late deaf CI recipients*: F(1.6, 14.4) = 33.6, p<0.001, effect size η_p_^2^ = 0.8; *MCCD*: F(1.4, 12.2) = 25.7, p<0.001, effect size η_p_^2^ = 0.7; *MCLD*: F(1.3, 11.8) = 22.0, p<0.001, effect size η_p_^2^ = 0.7), and for each group reaction times to bimodal stimuli were faster compared to unimodal stimuli (*congenitally deaf CI recipients*: F(1, 8) = 32.9, p<0.001, effect size η_p_^2^ = 0.8; *late deaf CI recipients*: F(1, 9) = 27.4, p = 0.001, effect size η_p_^2^ = 0.7; *MCCD*: F(1, 9) = 10.4, p = 0.01, effect size η_p_^2^ = 0.5; *MCLD*: F(1, 9) = 6.2, p = 0.03, effect size η_p_^2^ = 0.4).

In particular, for the MCCD the bimodal condition was faster compared to the tactile (t(9) = 8.9, p<0.001, d = 5.6) and the auditory condition (t(9) = 6.7, p<0.001, d = 4.2) while the two unimodal conditions did not differ (p = 0.7). Similarly, for the MCLD the bimodal condition was faster compared to the tactile (t(9) = 6.0, p<0.001, effect size d = 3.8) and the auditory condition (t(9) = 8.8, p<0.001, effect size d = 5.6) while the two unimodal conditions did not differ (p = 0.4).

On the contrary, for the congenitally deaf CI recipients, not only was the bimodal condition faster compared to the tactile (t(8) = 2.6, p = 0.03, effect size d = 1.7) and auditory condition (t(8) = 7.7, p<0.001, effect size d = 5.1), but also the two unimodal conditions differed (t(8) = 4.0, p = 0.004, effect size d = 2.7), in that this group responded faster to tactile than to auditory stimuli (251 ms (SE = 15) vs. 286 ms (SE = 14), respectively). Similarly, late deaf CI recipients showed faster reaction times for bimodal compared to the tactile (t(9) = 7.0, p<0.001, effect size d = 4.4) and auditory condition (t(9) = 8.1, p<0.001, effect size d = 5.1) and faster reaction times for tactile than auditory stimuli (309 ms (SE = 14) vs. 331 ms (SE = 11), respectively; t(9) = 2.3, p = 0.05, effect size d = 1.5).

In order to compare the two groups (congenitally and late deaf CI recipients), we ran two separate ANOVAS for each group matched with its control group (congenitally deaf CI vs. MCCD and late deaf CI vs. MCLD). The comparison between congenitally deaf CI recipients and MCCD revealed a main effect of condition (F(1.4, 23.1) = 45.7, p<0.001, effect size η_p_^2^ = 0.7) and a significant interaction between condition and group (F(1.4, 23.1) = 6.4, p = 0.01, effect size η_p_^2^ = 0.3) but no significant group effect (p = 0.2). Independent-samples t-tests showed that congenitally deaf CI were faster for unimodal tactile stimuli only compared to their matched controls (t(17) = 2.3, p = 0.03, effect size d = 1.1).

On the contrary, the comparison between late deaf CI recipients and their matched controls revealed a significant main effect of Condition (F(1.5, 27.8) = 50.6, p<0.001, effect size η_p_^2^ = 0.3) but only a marginally significant interaction between Condition and Group (F(1.5, 27.8) = 3.3, p = 0.06, effect size η_p_^2^ = 0.2). Moreover, independent samples t-tests showed no difference for any condition between groups.

To document whether faster responses to tactile stimuli are influenced by age at deafness onset, years of deafness, age at implantation or years of CI use, we correlated these factors with the mean response to unimodal tactile stimuli. To avoid confounds (i.e., our congenital and late deaf CI recipients have different characteristics, such as age, and age at implantation is statistically different between groups) only for age at deafness onset the correlation was made between both groups and their reaction times. For the other factors, analyses were kept separate. Note, that age at implantation and years of deafness have the same numerical value for congenitally deaf CI, so that disambiguating the role of these two factors is rather difficult.

**Figure 2:**
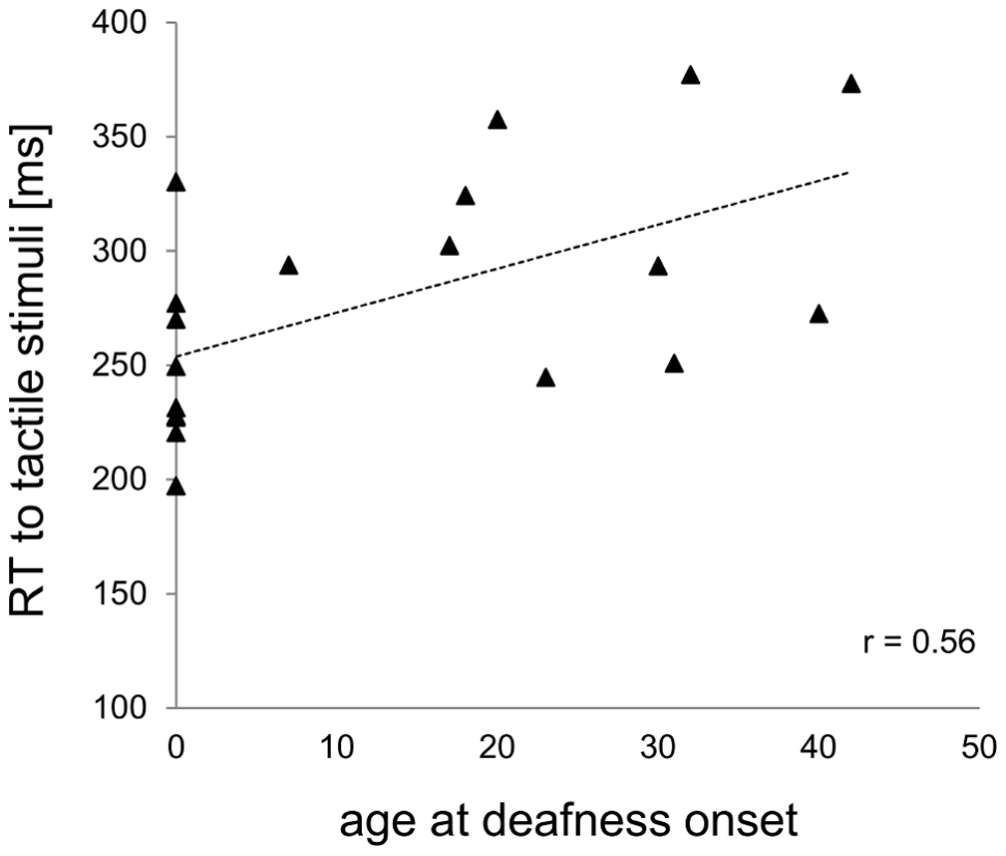
Correlation between unisensory tactile stimuli and age at deafness onset. Reaction times (in ms) to unisensory tactile stimuli as a function of age at deafness onset (in years).

Figure 2 shows that only age at deafness onset (r = 0.56, n = 19, p = 0.01) was a significant factor, while all others did not explain our results. However, it is worth noting that years of CI use showed a trend towards significance for the late deaf CI recipients (r = −0.57, n = 10, p = 0.09), in that reaction times to tactile stimuli tend to get faster the longer the CI recipients has worn his implant.

**Figure 3:**
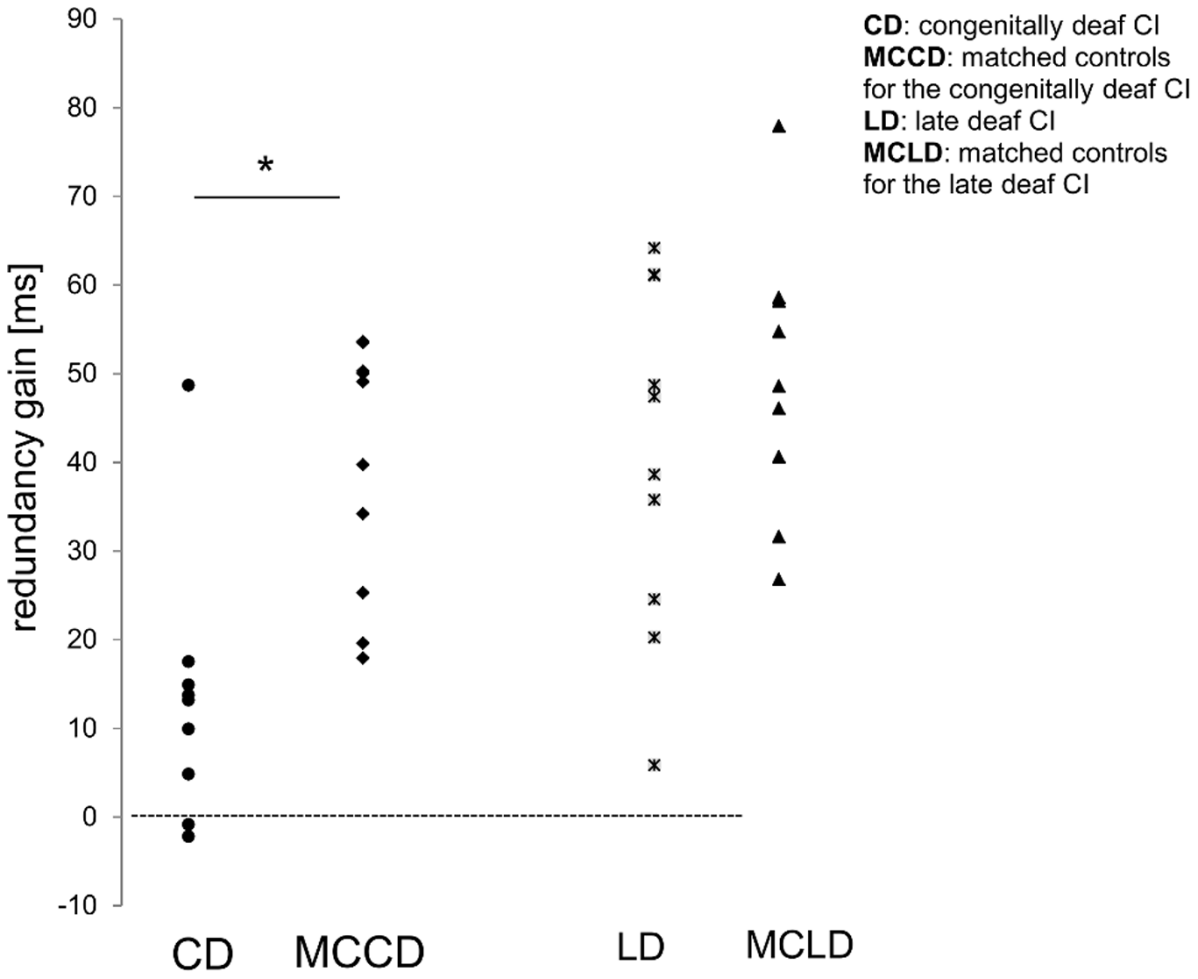
Redundancy gains (in ms). Redundancy gains for each participant (in ms), computed as the difference between the mean reaction times of bimodal stimuli and the fastest unimodal stimuli. Note that congenitally but not late deaf CI recipients showed lower redundancy gains compared to their matched controls.

**Figure 4:**
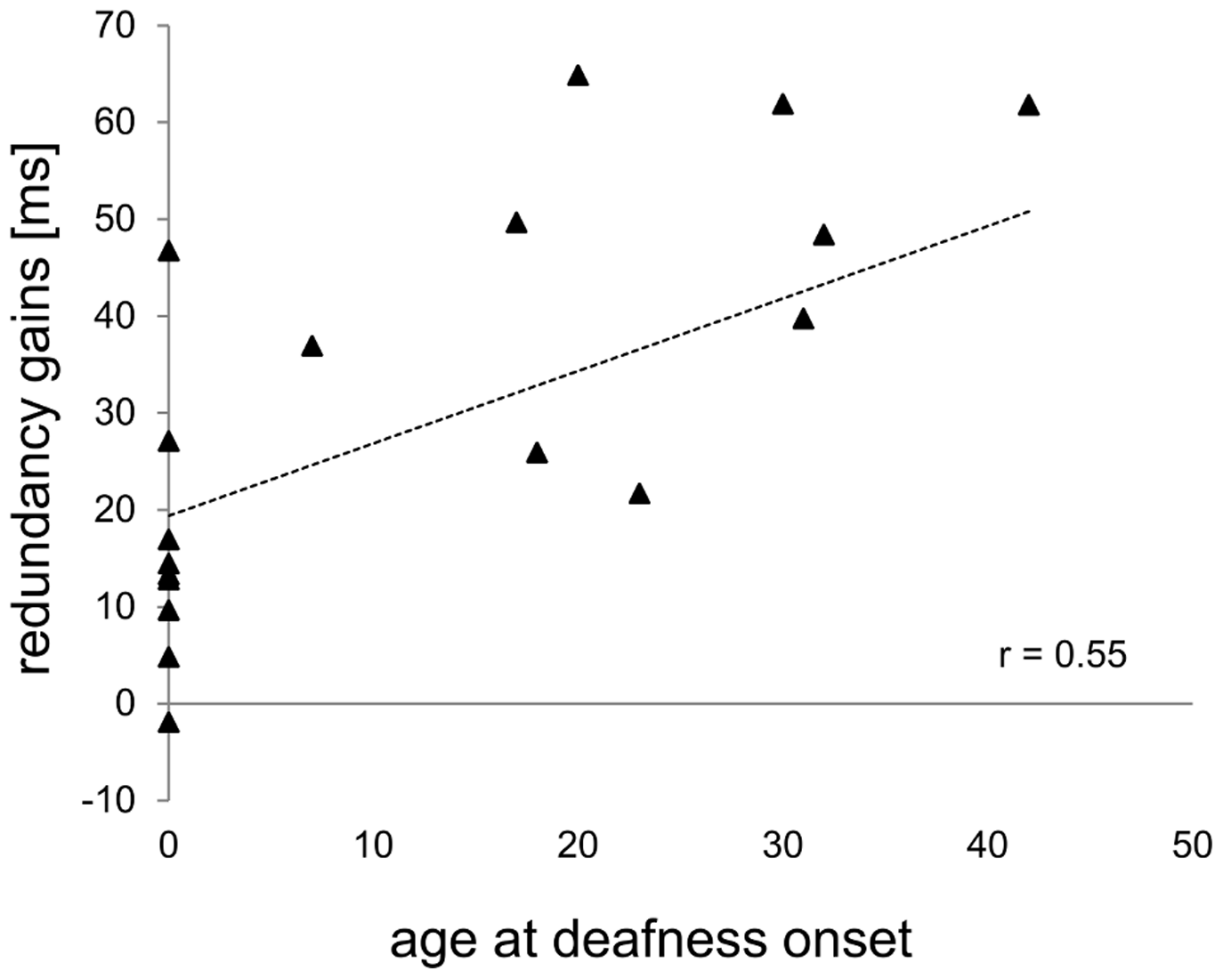
Correlation between redundancy gain and age at deafness onset. Redundancy gains (in ms) plotted as a function of age at deafness onset (in years).

Figure 3 shows redundancy gains for each participant, separately for group. Between groups analyses (congenitally deaf CI vs. MCCD and late deaf CI vs. MCLD) showed redundancy gains differences, specifically between congenitally deaf CI recipients and their matched controls (t(17) = 3.8, p = 0.001, effect size d = 1.8), caused by lower redundancy gains in congenitally deaf CI recipients compared to their matched controls (mean: 13 ms (SE = 4.5), vs. 38 ms (SE = 4.1), respectively). On the contrary, we found no difference between late deaf CI recipients and their matched controls. Furthermore, the correlations (see Fig. 4) proved significant for age at deafness onset only (r = 0.55, n = 19, p = 0.02).

To document whether there is a trade-off between redundancy gain and reaction times to unimodal tactile stimuli (i.e., shorter reaction times to tactile stimuli parallel lower multisensory gains), we correlated these two factors separately for congenitally and late deaf CI recipients. We found a significant correlation for the congenitally (r = 0.7, n = 9, p = 0.05) but not for the late deaf CI recipients (r = 0.6, n = 10, p = 0.07).

**Figure 5:**
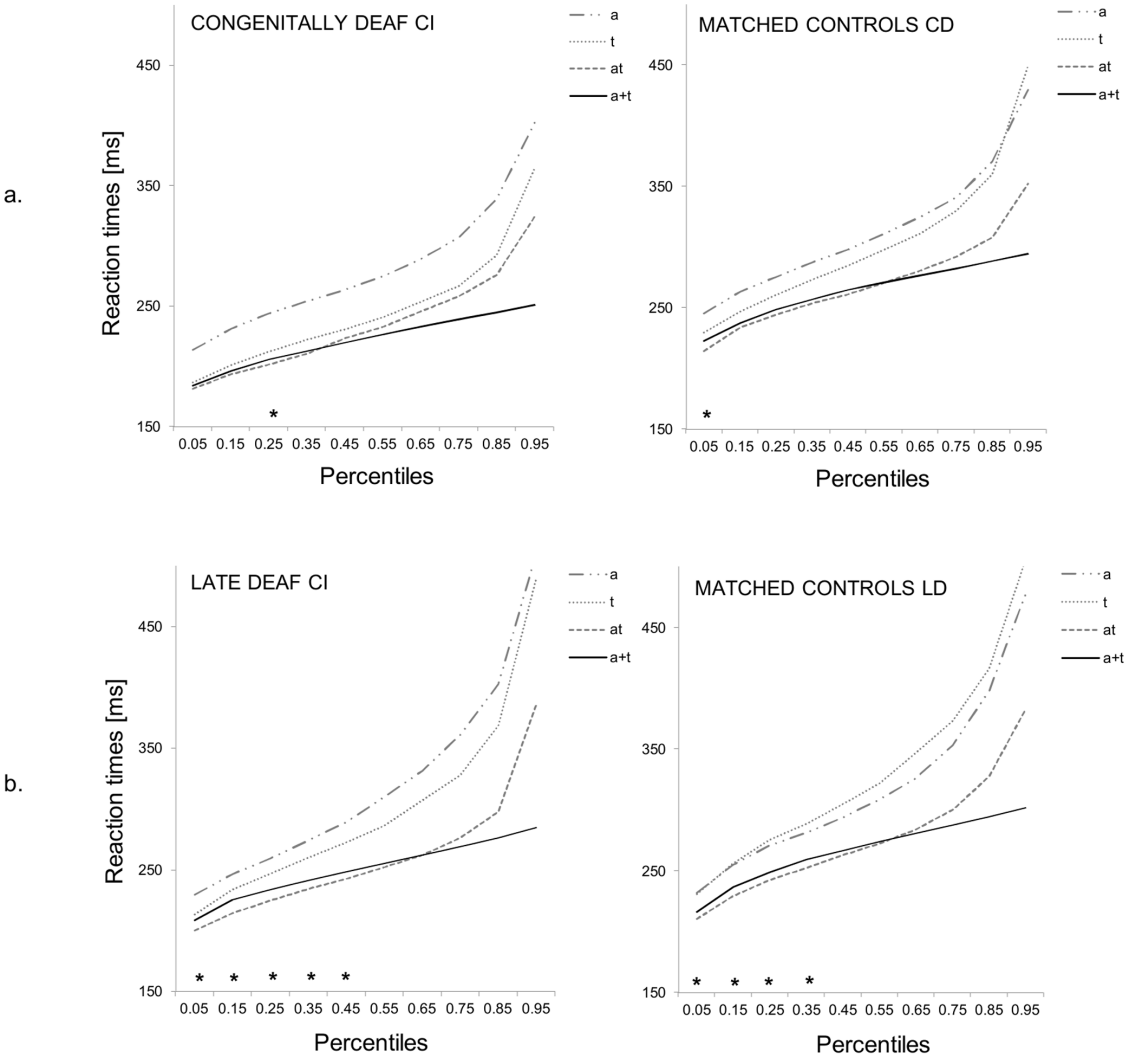
Multisensory facilitation indexed by violation of the race model. Cumulative distribution functions for response time to unisensory auditory and tactile stimuli and crossmodal stimuli for a. congenital deaf CI recipients and their age-matched controls and b. late deaf CI recipients and their age-matched controls. The filled black line indicates the summed proportions to unimodal stimuli (*a+t*, race model), *at* the violation of the race model, and *a* and *t* the responses to single auditory and tactile stimuli, respectively.

The race model inequality test revealed a significant violation of the race model assumption in all three groups (see Fig. 5). It is worth noting that the violation of the race model occurred in the third percentile for the MCCD, in the first 4 percentiles for the MCLD, in the first percentile for the congenitally deaf CI recipients and in the first five percentiles for the late deaf CI recipients. However, we found no difference in the amount of violation between groups. Moreover, the correlations between amount of violation and the factors of interest did not prove significant.

## Discussion

The present study examined whether congenitally and late deaf CI recipients are able to integrate simple audio-tactile stimuli as measured in a redundant target paradigm.

We found reliable redundancy gains for audio-tactile stimuli compared to unimodal stimuli and a violation of the race model for both congenitally and late deaf CI recipients. A violation of the race model is commonly accepted evidence for a true coactivation and thus crossmodal integration. The finding that not only late but congenitally deaf CI recipients were able to integrate audio-tactile stimuli as well suggests that, congenital sensory deprivation does not prevent the development of multisensory integration of simple synchronous stimuli, which is in line with [9], who tested congenital cataract individuals on the same paradigm as used in the present study. The similar results obtained in the two studies allow us speculating on the fact that crossmodal integration might build on broadly tuned innate abilities (i.e., detection of simultaneously presented multimodal information). However, it is worth discussing the differences between our results and [9]. Indeed, cataract patients [9] showed more similar multisensory gains with respect to their matched controls compared to our CI recipients (particularly the congenitally CI recipients), suggesting a more fully recovery in the cataract patients compared to our CI recipients. This may be due to the earlier sensory restoration performed in the cataract patients compared to our congenitally CI recipients, who received their CI on average in adulthood. Indeed, the earliest surgery in the deaf CI recipients was performed at a higher age (>2 years) than the latest cataract surgery in most of the patients studied in [9]. If the critical period for multisensory processes exists within the first about 2.5 years [11], it is not surprising that we did not find a statistically significant correlation between age at implant or years of CI experience and reaction times or redundancy gains.

It is worth considering why our results appear in conflict with [15], who used simple audio-tactile stimuli too and found no integration abilities for both congenitally and late deaf CI recipients, thus concluding that years of deafness, and not age at deafness onset, influence multisensory processing capabilities. First, it should be noted that the authors adopted a paradigm that tests the ability to fuse crossmodal incongruent information, while our task made use of congruent, synchronous stimuli. The lack of fusion of incongruent audio-tactile information could be explained by the enhanced tactile abilities of CI recipients as reported earlier for deaf individuals [14] and confirmed in the present study by showing shorter reaction times to tactile stimuli in the congenitally deaf CI recipients. Based on an optimal integration rule [22], higher tactile performance could result in higher reliance upon tactile information and thus less interference when incongruent auditory input is presented (see [16] for an analogous reasoning for lower audio-tactile fusion in the blind). Recently, it has been speculated as well that enhanced skills in the intact modality after sensory deprivation might originate from a reorganisation at the level of multisensory brain structures as a consequence of sensory deprivation and that these neural changes in turn would interfere with functional recovery [23], [24]. Our data do not allow disambiguating between these two alternatives. Both accounts are able to explain the positive correlation between redundancy gain and reaction times to unimodal tactile stimuli in congenitally deaf CI recipients only, namely participants who were faster at detecting unimodal tactile stimuli also had a lower multisensory gain. This leads to the discussion as to why congenitally deaf CI recipients showed lower redundancy gains compared to their matched controls. While it could be argued that this difference stems from less multisensory integrating abilities, it might similarly emerge as a consequence of congenitally deaf CI recipients responding faster to unimodal tactile stimuli compared to their matched controls (the redundancy gain was computed as a difference between bimodal stimuli and the faster of the two unisensory stimuli). The advantage in reaction times for unimodal tactile stimuli in the congenitally deaf CI recipients, as discussed above could be explained in terms of a reorganisation of the sensory systems, by which the remaining senses improve to compensate the sensory loss [25], [14]. Our results showed that faster responses to tactile stimuli are only significantly influenced by one factor, namely age at deafness onset, which suggests that this cross-modal reorganisation may decrease the later deafness is acquired in development. Furthermore, because for our congenitally deaf CI recipients other factors such as age at implantation and years of CI use were not found significant, it could be speculated that once cross-modal changes have taken place, they cannot be “re-wired” through auditory reafferentation, at least not after the age of 2 years. In support to this claim, several studies [32], [33], [34] have provided evidence that particularly deafened individuals with massive crossmodal take-over of auditory regions by visual input are less likely to benefit from implantation, thus questioning, for some specific cases, the potential of restoring hearing. Recently, [35] examined the relationship between crossmodal plasticity and speech perception in prelingually and postlingually deafened CI recipients by correlating the amplitude of visual evoked potential (VEP) over the right temporal lobe with the word perception scores of each CI recipient. The authors found that the amplitude of the VEP increased while the word perception scores decreased. Most importantly, this was observed in prelingually but not postlingually CI recipients, suggesting that, in accord with our findings, the influence of crossmodal plasticity on speech perception abilities depends upon deafness onset.

In conclusion, the present study provides evidence that basic audio-tactile stimuli can be integrated following congenital and late deafness after sensory restoration. We provide evidence for compensatory plasticity early in life, leading to an improvement in the tactile modality accompanied by lower multisensory gains following auditory loss.

## References

[1] Wallace MT, Stein BE (2007) Early experience determines how the senses will interact. J Neurophysiol 97: 921–926. 16914616 10.1152/jn.00497.2006

[2] Yu L, Rowland BA, Stein BE (2010) Initiating the development of multisensory integration by manipulating sensory experience. J Neurosci 30: 4904–4913. 20371810 10.1523/JNEUROSCI.5575-09.2010 PMC2858413

[3] Carriere BN, Royal DW, Perrault TJ, Morrison SP, William Vaughan J, et al (2007) Visual deprivation alters the development of cortical multisensory integration. J Neurophysiol 98: 2858–2867. 17728386 10.1152/jn.00587.2007

[4] Wallace MT, Perrault TJ, David Hairston P, Stein BE (2004) Visual experience is necessary for the development of multisensory integration. J Neurosci 24: 9580–9584. 15509745 10.1523/JNEUROSCI.2535-04.2004 PMC6730167

[5] Putzar L, Goerendt I, Lange K, Rösler F, Röder B (2007) Early visual deprivation impairs multisensory interactions in humans. Nat Neurosci 10: 1243–1245. 17873871 10.1038/nn1978

[6] Lewkowicz DJ, Ghazanfar AA (2009) The emergency of multisensory systems through perceptual narrowing. Trends Cogn Sci 13: 470–478. 19748305 10.1016/j.tics.2009.08.004

[7] Lewkowicz DJ, Ghazanfar AA (2006) The decline of cross-species intersensory perception in human infants. P Natl Acad Sci U S A 103: 6771–6774. 10.1073/pnas.0602027103 PMC1458955 16618919

[8] Putzar L, Hötting K, Röder B (2010) Early visual deprivation affects the development of face recognition and of audio-visual speech perception. Restor Neurol Neurosci 28: 251–257. 20404412 10.3233/RNN-2010-0526

[9] Putzar L, Gondan M, Röder B (2012) Basic multisensory functions can be acquired after congenital visual pattern deprivation in humans. Dev Neuropsychol 37: 697–711. 23145567 10.1080/87565641.2012.696756

[10] Tremblay C, Champoux F, Lepore F, Théoret H (2010) Audiovisual fusion and cochlear implant proficiency. Restor Neurol Neurosci 28: 283–291. 20404415 10.3233/RNN-2010-0498

[11] Schorr E, Fox NA, van Wassenhove V, Knudsen E (2005) Auditory-visual fusion in speech perception in children with cochlear implants. P Natl Acad Sci U S A 102: 18748–18750. 10.1073/pnas.0508862102 PMC1317952 16339316

[12] Gilley PM, Sharma A, Mitchell TV, Dorman MF (2010) The influence of a sensitive period for auditory-visual integration in children with cochlear implants. Restor Neurol Neurosci 28: 207–218. 20404409 10.3233/RNN-2010-0525 PMC3684694

[13] Pavani F, Röder B (2012) Crossmodal plasticity as a consequence of sensory loss: insights from blindness and deafness. In: Stein B, editor. The New Handbook of Multisensory Processes. Cambridge, MIT Press. 737–760.

[14] Levänen S, Hamdorf D (2001) Feeling vibrations: enhanced tactile sensitivity in congenitally deaf humans. Neurosci Lett 301: 75–77. 11239720 10.1016/s0304-3940(01)01597-x

[15] Landry SP, Guillemot JP, Champoux F (2013) Temporary deafness can impair multisensory integration. A study of cochlear implant users. Psychol Sci 24: 1260–1268. 23722977 10.1177/0956797612471142

[16] Hötting K, Röder B (2004) Hearing cheats touch, but less in congenitally blind than in sighted individuals. Psychol Sci 15: 60–64. 14717833 10.1111/j.0963-7214.2004.01501010.x

[17] Rouger J, Lagleyre S, Fraysse B, Deguine O, Barone P (2007) Evidence that cochlear-implanted deaf patients are better multisensory integrators. P Natl Acad Sci U S A 105: 7295–7300. 10.1073/pnas.0609419104 PMC1855404 17404220

[18] Miller J (1982) Divided attention: evidence for coactivation with redundant targets. Cognitive Psychol 14: 247–279. 10.1016/0010-0285(82)90010-x 7083803

[19] Raab DH (1962) Statistical facilitation of simple reaction times. T New York Acad Sci 24: 574–590. 10.1111/j.2164-0947.1962.tb01433.x 14489538

[20] Ulrich R, Miller J, Schröter H (2007) Testing the race model inequality: an algorithm and computer programs. Behav Res Methods 39: 291–302. 17695357 10.3758/bf03193160

[21] Colonius H, Diederich A (2006) The race model inequality: a geometric measure of the amount of violation. Psychol Rev 113: 148–154. 16478305 10.1037/0033-295X.113.1.148

[22] Ernst MO, Banks MS (2002) Humans integrate visual and haptic information in a statistically optimal fashion. Nature: 415, 429–433. 10.1038/415429a 11807554

[23] Lewkowicz D, Röder B (2011) Development of multisensory processes and the role of early experience. In: Stein B, editor. The New Handbook of Multisensory Processes. MIT Press, Cambridge/MA, USA.

[24] Lomber SG, Meredith MA, Kral A (2012) Cross-modal plasticity in specific auditory cortices underlies visual compensations in the deaf. Nat Neurosci 13: 1421–1427. 10.1038/nn.2653 20935644

[25] Levänen S, Jousmäki V, Hari R (1998) Vibration-induced auditory-cortex activation in a congenitally deaf adult. Curr Biol 8: 869–872. 9705933 10.1016/s0960-9822(07)00348-x

[26] Gondan M, Lange K, Röder B (2004) The redundant target effect is affected by modality switch costs. Psychon B Rev 11: 307–313. 10.3758/bf03196575 15260198

[27] Occelli V, Spence C, Zampini M (2011) Audiotactile interactions in temporal perception. Psychon B Rev 18: 429–454. 10.3758/s13423-011-0070-4 21400125

[28] Slater A, Kirby R (1998) Innate and learned perceptual abilities in the newborn infant. Exp Brain Res 123: 90–94. 9835396 10.1007/s002210050548

[29] Kuhl PK, Stevens E, Hayashi A, Deguchi T, Kiritani S, et al (2006) Infants show a facilitation effect for native language phonetic perception between 6 and 12 months. Dev Sci 9: F13–F21. 16472309 10.1111/j.1467-7687.2006.00468.x

[30] Amedi A, Von Kriegstein K, Van Atteveldt NM, Beauchamp MS, Naumer MJ (2005) Functional imaging of human crossmodal identification and object recognition. Exp Brain Res 166: 559–571. 16028028 10.1007/s00221-005-2396-5

[31] Lewkowicz DJ (2000) The development of intersensory temporal perception: an epigenetic systems/limitations view. Psychol Bull 126: 281. 10748644 10.1037/0033-2909.126.2.281

[32] Sandmann P, Dillier N, Eichele T, Meyer M, Kegel A, et al (2011) Visual activation of auditory cortex reflects maladaptive plasticity in cochlear implant users. Brain 135: 555–568. 10.1093/brain/awr329 22232592

[33] Lee DS, Lee JS, Oh SH, Kim SK, Kim JW, et al (2001) Cross-modal plasticity and cochlear implants. Nature 409: 149–150. 10.1038/35051653 11196628

[34] Lee HJ, Giraud AL, Kang E, Oh SH, Kang H, et al (2007) Cortical activity at rest predicts cochlear implantation outcome. Cereb Cortex 17: 909–917. 16731883 10.1093/cercor/bhl001

[35] Buckley KA, Tobey EA (2011) Cross-modal plasticity and speech perception in pre- and postlingually deaf cochlear implant users. Ear Hearing 32: 2–15. 20829699 10.1097/AUD.0b013e3181e8534c

